# Mapping Quantitative Trait Loci Associated With Resistance to Aflatoxin Accumulation in Maize Inbred Mp719

**DOI:** 10.3389/fmicb.2020.00045

**Published:** 2020-02-04

**Authors:** Erika D. Womack, W. Paul Williams, Gary L. Windham, Wenwei Xu

**Affiliations:** ^1^Corn Host Plant Resistance Research Unit, USDA-ARS, Mississippi State, MS, United States; ^2^Texas A&M AgriLife Research, Lubbock, TX, United States

**Keywords:** quantitative trait locus, maize, *Aspergillus flavus*, aflatoxin, host-plant resistance

## Abstract

Aflatoxins are carcinogenic and toxic compounds produced principally by fungal species *Aspergillus flavus* (Link: Fries) and *A. parasiticus* (Speare), which are common contaminants of food and feed. Aflatoxins can be found at dangerously high levels and can readily contaminate pre-harvest maize (*Zea mays* L.) grain. Sources of resistance to aflatoxin accumulation in maize have been identified, however, the highly quantitative nature and complex inheritance of this trait have limited the introgression of aflatoxin accumulation resistance into agronomically desirable lines. Mapping of quantitative trait loci (QTL) was performed on a bi-parental population comprised of 241 F2:3 families derived from the cross of inbred lines Mp705 (susceptible) × Mp719 (resistant). The mapping population was phenotyped in replicated field trials in three environments for resistance to aflatoxin accumulation under artificial inoculation with an *A. flavus* spore suspension. The genetic linkage map was constructed with 1,276 single nucleotide polymorphism (SNP) and simple sequence repeat (SSR) molecular markers covering a total genetic distance of 1,642 cM across all ten maize chromosomes. Multiple interval mapping revealed that majority of the aflatoxin-reducing alleles and the source for the larger effect QTL identified in this study were contributed from Mp719, the resistant parent. Two QTL identified on chromosome 1 (bin 1.06–1.07) and chromosome 3 (bin 3.09) were the most stable across different environments and when combined, explained 24.6% of the total phenotypic variance across all three environments. Results from the study showed that these chromosomal regions harbor important QTL for influencing aflatoxin accumulation, which is consistent with previous reports with other different mapping populations. These stable QTL were the most promising for controlling aflatoxin accumulation in maize grain. Identifying beneficial alleles derived from Mp719 and closely linked molecular markers through QTL analysis for implementation of MAS could accelerate breeding efforts to reduce aflatoxin accumulation in maize.

## Introduction

Maize (*Zea mays* L.), cultivated worldwide, is an agronomically important grain crop that plays a significant role in food security. Global maize production in 2017 totaled 1.1 billion tons (FAOSTAT, 2019) and the United States, the largest producer of maize, produced over 30% of the total world supply and contributed about $52 billion to the economy (FAOSTAT, 2019; National Corn Growers Association [NCGA], 2019). Abiotic and biotic stresses pose a serious threat to maize production that can lead to major yield losses and diminished grain quality causing significant impacts to the economy and threatening the livelihood of millions. The most important “biotic stresses on maize are primarily pathogens” (Gong et al., 2014). *Aspergillus*, an ear-rot fungal pathogen that produces mycotoxin, is especially problematic as the mycotoxin can be present at dangerously high levels in the grain with or without fungal growth (Thompson and Raizada, 2018). Discovered nearly 60 years ago, aflatoxin, a type of mycotoxin, has become known as a common contaminant of animal feed and human food supply. Aflatoxins are highly toxic secondary metabolites produced mainly by fungal species *Aspergillus flavus* (Link: Fries) and *A. parasiticus* (Speare) (Lin and Dianese, 1976). In developing countries, most households consume crops that they produce including maize and as a result, may be chronically exposed to aflatoxin (Strosnider et al., 2006). Regrettably, aflatoxins are linked to many negative health consequences that occur from consumption of contaminated food, including immunosuppression, teratogenic and carcinogenic effects (Bennett and Klich, 2003).

Many countries worldwide have established regulatory guidelines for maximum tolerable levels of food and feed to minimize exposure to aflatoxin. The United States Food and Drug Administration (FDA) imposed action levels for human food and animal feed. The safety range for aflatoxin in foodstuff is 20 and 0.5 ng g^–1^ for milk. To maintain a safe level of contaminants in feed for breeding animals, the action level is 100–200 ng g^–1^ (US Food and Drug Administration [FDA], 2000). The regulation of aflatoxin contamination is associated with economic losses that include the cost of preventative and mitigation measures using inspections, sampling, and analyses; the reduced value or disposal of contaminated food and feed; and losses caused by the reduction in animal productivity (Wu, 2015). Maize is one of the major crops with a high economic risk for aflatoxin contamination resulting in an estimated loss of up to $225 million annually in the United States (Wu, 2006), and as much as a billion dollars globally (Mitchell et al., 2016). Aflatoxin contamination has historically been a recurrent annual problem in the southern and southeastern regions of the United States (Mitchell et al., 2016). Regulatory guidelines to reduce the risk of aflatoxin exposure have little relevance in many food-insecure populations where they often rely on maize for daily nutrition and income (Strosnider et al., 2006).

Pre-harvest management is a critical practice for minimizing aflatoxin contamination of maize (Mahuku et al., 2019). Resistance to aflatoxin accumulation can be achieved by reducing the fungal infection in the grain, reducing the amount of toxin produced by the fungus, or both (Warburton et al., 2011). Strategies including development of acceptable agronomic practices, insect management, and biological control have been exploited to improve maize resistance to aflatoxin accumulation (Widstrom, 1996; Atehnkeng et al., 2008). Native host-plant resistance through conventional breeding strategies offers a more attractive, safe, and cost-effective solution for controlling aflatoxin production in maize (Brown et al., 1999). Most of the known resistance to aflatoxin accumulation in maize has been found in a limited number of temperate and several sources of tropical germplasm. Early breeding efforts identified *A. flavus*-resistant inbred lines Mp420 and Mp313E (Scott and Zummo, 1990, 1992). Additional germplasm lines developed for resistance to aflatoxin accumulation included GT-601–GT-603 developed from the GT-MAS:gk population, and Mp715, Mp717–Mp719, Tx736, Tx739, Tx740, Tx772, TZAR101–TZAR106 (McMillian et al., 1993; Williams and Windham, 2001, 2006, 2012; Llorente et al., 2004; Guo et al., 2007, 2011; Menkir et al., 2008; Mayfield et al., 2012). Despite steady development of genetic sources of resistance to aflatoxin accumulation in maize germplasm, attempts to transfer resistance to more agronomically adequate hybrids have been challenging (Abbas et al., 2002; Warburton and Williams, 2014). The identification of quantitative trait loci (QTL) and the discovery of associated molecular markers to facilitate the transfer of beneficial alleles to elite lines through marker-assisted selection (MAS) could contribute to traditional breeding efforts (Jiang, 2013).

Many studies have investigated genomic regions using QTL analysis for maize resistance to aflatoxin accumulation to identify molecular markers for use in MAS (Paul et al., 2003; Widstrom et al., 2003; Brooks et al., 2005; Alwala et al., 2008; Warburton et al., 2009, 2011; Willcox et al., 2013; Yin et al., 2014; Dhakal et al., 2016; Smith et al., 2019). One germplasm line, Mp719, released as a source of resistance to aflatoxin accumulation, has not been previously mapped for aflatoxin accumulation and is included in the present study. The objective of this study is to identify QTL associated with the reduction of aflatoxin accumulation in maize in a bi-parental mapping population, comprising 241 F2:3 families derived from a cross between Mp719 (resistant) and Mp705 (susceptible).

## Materials and Methods

### Population Development

The mapping population was derived from a cross between maize inbred lines, Mp719 and Mp705. Mp719 (PI 662046) is a breeding inbred line that was developed and released by the USDA’s Agricultural Research Service (USDA-ARS, Mississippi) as a source of resistance to aflatoxin accumulation (Williams and Windham, 2012). During the trial performed by Williams and Windham (2012), Mp719 had a geometric mean of 74 ng g^–1^ and the means for the susceptible checks in the experiment were 1,153 (Va35), 3,452 (Ga209), and 5,615 ng g^–1^ (SC212m). Mp719 was developed from a cross between inbred lines Mp715 and Va35. Mp715 (PI 614819) was released as a source of resistance for aflatoxin accumulation and developed from Tuxpan, an open-pollinated Southern Dent derived from the Mexican dent germplasm, Tuxpeño (Williams and Windham, 2001). Va35 (PI 587150) was developed and released by the Virginia Agricultural Experiment Station by self-pollination of the backcross, (C103 x T8)T8 (Gracen, 1986). Susceptible to many diseases including aflatoxin accumulation but with good agronomic qualities, Va35 is a non-stiff stalk southern United States maize inbred line derived from Lancaster Surecrop. Mp705, the susceptible parent of the mapping population used in this study, was released in 1984 (registration number GP-130) by the USDA-ARS as a source of resistance to insect leaf-feeding damage (Williams and Davis, 1984). Mp705 was derived from MpSWCB-4, a population that was developed as a source of resistance to leaf-feeding damage caused by southwestern corn borer (SWCB), *Diatraea grandiosella* (Dyar) (Scott and Davis, 1981).

Pre-harvest aflatoxin contamination field trials were planted in three environments: 2017 and 2018 at the R. R. Foil Plant Science Research Center, Mississippi State, Mississippi (MS) and 2017 at the Quaker Research Farm, Texas A&M AgriLife Extension Center, Lubbock, Texas (TX). The population was developed by self-pollinating the F1 plant to produce F2 seed and the F2 plants were self-pollinated to produce 241 F2:3 families. The tests were phenotyped for aflatoxin accumulation in three replications and included 241 F2:3 families, the inbred parents, Mp719 and Mp705, and their F1 hybrid. This mapping population was also phenotyped, in a separate field experiment, for fall armyworm *Spodoptera frugiperda* leaf-feeding damage by Womack et al. (2020). Experiments were sown in a single 5.1 m long row plot spaced 0.96 m apart and thinned to 20 plants in a randomized complete block design. Plants were maintained with local standard cultural practices and irrigation.

### Inoculum Preparation and Phenotyping

Inoculum was prepared from the *A. flavus* isolate NRRL 3357, which is known to produce aflatoxin in maize grain (Windham and Williams, 1999, 2002). The fungal inoculum was increased on 40 mesh, sterilized corn-cob grits (Grit-O-Cobs, The Andersons Inc., Maumee, OH, United States) in 500 mL flasks, each containing 50 g of grits and 100 mL of sterile, distilled water, and incubated at 28°C for 3 weeks. The conidia were washed from the grits using 500 mL sterile distilled water containing 20 drops L^–1^ of Tween 20 (Sigma-Aldrich, St. Louis, MO, United States) and filtered through four layers of sterile cheesecloth. Concentrations of conidia were determined with a hemacytometer and diluted with sterile, distilled water to 9 × 107 *A. flavus* conidia mL^–1^. Inoculum not immediately used was stored at 4°C. Developing ears were inoculated using the side-needle technique according to Zummo and Scott (1989). Seven days after silks had emerged from 50% of the 20 plants in a plot, the top ear of each plant was inoculated with the *A. flavus* spore suspension. Using an Idico tree-marking gun fitted with a 14-gauge needle, a 3.4 mL suspension containing 3 × 108 *A. flavus* conidia was injected through the husks into the side of the ear. There was an approximate range of 14 days between the first set of genotypes that flowered until the last set of genotypes that flowered in every year. The inoculated ears in each plot were hand-harvested in bulk approximately 60 days after inoculation of each plot and dried at 53°C for 7 days. Ears were mechanically shelled and the grain was mixed thoroughly before grinding with a Romer subsampling mill (Union, MO, United States). The concentration of aflatoxin from a 50 g ground sample was determined using VICAM AflaTest (Watertown, MA, United States) per manufacturer’s instructions.

### Statistical Analysis of Phenotypic Data

The concentrations for aflatoxin were log-transformed to convert skewed data to conformed, normalized values using ln(y + 1), where y is the concentration of the aflatoxin in a sample. The three check genotypes (Mp705, Mp719, and the F1 hybrid) of the experiment were analyzed in SAS 9.4 (SAS Institute, 2014, Cary, NC, United States). The genotypes were subjected to analysis of variance (ANOVA) using PROC MIXED. Genotype, environment (location and year), and genotype-by-environment interaction were the fixed effects of the model and block nested in environment was the random effect.

The best linear unbiased predictors (BLUPs) for each F2:3 family (genotype) mean were calculated using the PROC MIXED function in SAS 9.4 (SAS Institute, 2014, Cary, NC, United States). Genotype and block were estimated as random effects of the model within an environment. When combined over all environments, genotype, environment (location and year), and genotype-by-environment interaction were the fixed effects of the model and block nested in environment was the random effect. The BLUP value of each family mean was used for QTL analysis. Estimates of the variance components of the F2:3 family means were obtained with restricted maximum likelihood (REML). Family mean broad-sense heritability (h2) estimates, within and across environments, were calculated according to Holland et al. (2003).

### Genotyping and QTL Mapping

The genotyping and QTL analysis used in this study have been previously described in detail by Womack et al. (2020). Briefly, leaf tissue samples were collected from the all F2:3 plants and the check genotypes and DNA was extracted from each sample using a modified cetyltrimethylammonium bromide (CTAB) method described by Saghai-Maroof et al. (1984). For genotyping analysis, SSR and SNP markers were run on the extracted DNA of the mapping population and were used for genetic linkage mapping construction in the JoinMap 4.0 (Van Ooijen, 2006) computer program. The dataset consisted of a total of 1,276 molecular markers, 1,247 SNP and 29 SSR markers, run on 241 F2:3 families. Markers were omitted from analysis if data was missing (>10%), if markers disrupted marker order and significantly deviated from the Mendelian 1:2:1 ratio, if markers were identical to other markers, and if a marker had a strong linkage outside of its own group. The recombination frequencies were converted to genetic distances (centiMorgans, cM) using the Haldane (1919) mapping function.

Quantitative trait loci analysis was conducted using Windows QTL Cartographer v. 2.5 software (Wang et al., 2012). A MIM preferred model of each environment was selected according to guidelines of Silva et al. (2012). Briefly, composite interval mapping (CIM) was performed to initiate model terms for multiple interval mapping (MIM) (Kao and Zeng, 1997; Kao et al., 1999). Main effect QTL were searched, and significant terms were added to the model only if the Bayesian information criterion (BIC) decreased. The interactions between main effect QTL were searched. Epistasis was identified when there was a significant interaction between two QTL. The position of the QTL was optimized after significant terms were added to the model. The process was repeated until no additional parameters could be added. Quantitative trait loci position and genetic effects (additive, dominance, and epistasis) were estimated and the observed phenotypic variance was obtained (Kao et al., 1999). The signs of the genetic effect of the QTL in a model were used to identify the origin of the aflatoxin-reducing alleles (Lübberstedt et al., 1997).

## Results

### Phenotypic Performance of Parental Lines and F2:3 Families

The resistant parent, Mp719, the susceptible parent, Mp705, and their F1, were planted as checks in each environment. There were many missing plots in TX 2017; therefore, this data was not included in analyses. When the data was combined across MS 2017 and MS 2018 environments, the ANOVA results indicated that block nested in environment (*p* = 0.1234) and the variability due to the interaction between genotype and environment (*p* = 0.1854) were not significant sources of variance (Table 1). Genotype (*p* = 0.0002) and environment (*p* = 0.0438), treated as main effects, were shown to have a significant effect on aflatoxin production. When analyzed for total aflatoxin accumulation, the resistant parent (Mp719) had significantly lower aflatoxin levels than the susceptible parent (Mp705) (*p* < 0.05) but, did not significantly differ from the F1 in MS 2017 and MS 2018 (*p* > 0.05) (Table 2).

**TABLE 1:** ANOVA results of the three check genotypes across two environments.

**Source of variance**	**df**	**Mean square**	***F* value**	***P* value**
Genotype	2	8.89	28.45	0.0002
Environment	1	6.67	8.46	0.0438
Genotype × environment	2	0.65	2.10	0.1854
Block (environment)	4	0.79	2.52	0.1234
Residual	8	0.31		

**TABLE 2:** Multiple comparisons of the mean aflatoxin concentrations of the check genotypes.

**MS 2017**			**MS 2018**		
	**lnAF^†^**	**Aflatoxin^‡^ (ng g^–1^)**		**lnAF**	**Aflatoxin (ng g^–1^)**
Mp705	5.86^a^	353.53	Mp705	6.32^a^	560.00
Mp719	3.20^b^	30.27	Mp719	4.83^b^	129.70
F1	3.17^b^	37.17	F1	4.72^b^	123.33

*^†^lnAF = log (total aflatoxin concentration + 1). ^‡^Mean concentration of aflatoxin followed by the same letter are not significantly different (α = 0.05).*

The mean aflatoxin concentrations for the F2:3 families were lower in the MS 2017 environment at a value of 47.1 ± 3.5 ng g^–1^ ($\bar{\text{x}}$ ± s.e.) than in the MS 2018 (172.2 ± 8.7 ng g^–1^) and TX 2017 (181.6 ± 9.9 ng g^–1^) environments (Table 3). The means of the transformed data of aflatoxin concentrations for the F2:3 families in each environment, calculated as BLUPs, were 2.56 ± 0.07 (MS 2017), 4.19 ± 0.06 (MS 2018), and 4.15 ± 0.07 (TX 2017) (Table 3). Variance components were estimated using REML and used to calculate heritability estimate across all environments (Table 4). The broad-sense heritability combined across the three environments was 0.56, and within each environment the repeatability estimates ranged from 0.43 to 0.54.

**TABLE 3:** Phenotypic descriptive statistics for the raw and log transformed aflatoxin values of the F2:3 families by year.

**Env**	**N Obs**	**Variable**	**Mean**	**Std error**	**Min**	**Median**	**Max**	**Std dev**	**CV**
MS 2017	720	Aflatoxin (ng g^–1^)	47.15	3.46	0.00	15.00	960.00	91.83	194.75
		lnAF^†^	2.56	0.07	0.00	2.77	6.87	1.78	69.73
MS 2018	720	Aflatoxin (ng g^–1^)	172.25	8.67	0.00	82.00	1720.00	230.16	133.62
		lnAF^†^	4.16	0.06	0.00	4.42	7.45	1.70	41.01
TX 2017	720	Aflatoxin (ng g^–1^)	181.62	9.93	0.00	90.00	1840.00	262.22	144.38
		lnAF^†^	4.11	0.07	0.00	4.51	7.52	1.84	44.70

*^†^lnAF = log (total aflatoxin concentration + 1). Env, environment; N Obs, number of observations; Std Error, standard error; Min, minimum; Max, maximum; CV, coefficient of variability; MS, Mississippi; TX, Texas.*

**TABLE 4:** Restricted maximum likelihood (REML) analysis used to estimate variance components of the F2:3 families by year and across all environments.

**Variance components**	**All environments**	**Variance components**	**MS 2017**	**MS 2018**	**TX 2017**
Genotype	0.47	Genotype	0.89	0.82	0.75
Environment	0.81	Block	0	0	0.04
Genotype x environment	0.35	Error	2.29	2.09	2.59
Block (env)	0.01				
Error	2.33				

*MS, Mississippi; TX, Texas.*

### Linkage Map Construction

The linkage map was constructed as in Womack et al. (2020) which included 1,276 SSR and SNP markers that resolved all maize chromosomes. The 10 linkage groups, corresponding to the 10 maize chromosomes, were identified with a LOD score of 3.0 and remained associated even at LOD = 10.0. There were 40 to 233 markers per linkage group (Supplementary Table S1). These markers spanned a total genetic distance of 1,642 cM with an average interval between markers of 1.3 cM and the largest interval was 16.8 cM. Chromosome 3 displayed some significant segregation distortion (α = 0.05) from bin 3.00 to 3.06, with markers deviating from the expected 1:2:1 Mendelian ratio. In this region, the allele from Mp719 appeared more frequently than the allele contribution from Mp705.

### QTL Analysis

Initial MIM models were assembled based on the models obtained from CIM analysis (Supplementary Table S2). Using MIM, a stepwise search for QTL identified genetic models containing six QTL and two epistatic interactions in the MS 2017 environment; four QTL and one epistatic interaction in MS 2018; and four QTL in TX 2017. When the data was combined over all environments, seven QTL and three interactions were identified (Table 5). Quantitative trait loci were identified on chromosomes 1, 3, 5, 8, and 10 in MS 2017; chromosomes 1, 2, 3, and 9 in MS 2018; and 1, 3, 4, and 6 in TX 2017. When combined over all environments, QTL were identified on chromosomes 1, 2, 3, 5, 8, and 10. The MIM models explained 42.3% (MS 2017), 27.4% (TX 2017) and 24.6% (MS 2018) phenotypic variances within each environment and 53.6% when combined overall environments. Four major QTL accounted for more than 10% of the total phenotypic variance in MS 2017 (11.3%, bin 1.06), TX 2017 (12.7%, bin 1.07), MS 2018 (10.5%, bin 3.09) and when combined across all environments (15.4%, bin 1.06).

**TABLE 5:** Multiple interval mapping results within and across environments.

**Env^∗^**	**Chr bin**	**QTL peak position**	**2-LOD interval**	**Marker^§^**	**Effect**		**Phenotypic variance**		
					**A^†^**	**D^‡^**	**A**	**D**	**Total**
		cM					%		
						
MS 2017	1.06	124.4	114.4–130.9	PZE-101154189	–0.294	0.021	11.3	0.0	11.3
	1.10	183.2	179.1–185.4	PZA00364-2	–0.258	–0.171	7.6	1.2	8.8
	3.09	156.7	151.3–161.8	PZE-103165542	–0.226	–0.091	5.6	0.3	5.9
	5.01	31.6	22.8–39.7	PHM3137-17	0.161	–0.141	2.4	1.1	3.5
	8.01	12.6	6.1–14.5	PZE-108002941	–0.154	0.195	1.6	2.3	3.9
	10.04	44.2	29.3–55.0	PZE-110067110	–0.198	0.141	4.5	0.6	5.1
	1.10 × 8.01				0.241 (AA)		2.4		2.4
	3.08 × 10.04				0.215 (AA)		1.4		1.4
									**42.3**
MS 2018	1.06	116.6	103.7–130.9	PHM5622-21	–0.180	0.145	3.9	0.9	4.8
	2.05	85.7	85.7–85.7	PHM4880-179	–0.148	0.238	2.2	2.2	4.4
	3.09	161.4	155.4–169.4	PZE-103169160	–0.304	–0.090	10.3	0.2	10.5
	9.04	63.0	59.3–63.6	PZE-109061001	0.024	–0.256	0.1	3.2	3.3
	1.06 × 3.09				0.214 (AA)		1.6		1.6
									**24.6**
TX 2017	1.07	141.4	138.9–147.2	PZE-101171655	–0.304	–0.095	12.6	0.1	12.7
	3.07	128.0	110.8–138.4	PZE-103144159	–0.186	0.032	4.7	0.2	4.9
	4.07	101.2	93.1–111	PZE-104101251	–0.197	–0.099	4.6	0.3	4.9
	6.04	49.6	31.6–55.9	PZE-106057733	–0.151	0.122	3.6	1.3	4.9
									**27.4**
Combined									
	1.06	118.6	104.6–123.3	PZE-101145417	–0.274	0.085	14.7	0.7	15.4
	1.09	177.9	173.9–181.8	PZE-101210110	–0.151	–0.117	5.4	1.4	6.8
	2.03	59.0	55.5–61.7	PZE-102036053	–0.139	0.113	2.8	0.8	3.6
	3.09	159.9	153.5–164.1	PZE-103169160	–0.209	–0.047	9.2	0.0	9.2
	5.00	3.0	0.0–17.7	PZE-105002166	0.141	0.006	4.1	0.0	4.1
	8.03	72.5	66.7–87.5	PZE-108057442	–0.102	–0.156	1.3	2.5	3.8
	10.06	78.6	64.6–92.8	PZE-110091181	–0.177	0.079	5.1	0.4	5.5
	1.09 × 2.03				−0.305 (DD)		1.5		1.5
	3.09 × 5.00				−0.301 (DD)		1.9		1.9
	1.09 × 8.03				0.167 (AA)		1.8		1.8
									**53.6**

*^∗^Environments: MS, Mississippi; TX, Texas. ^§^Marker closely associated with QTL peak position. ^†^Negative additive QTL effect indicate Mp719 is the source of the beneficial (aflatoxin-reducing) allele, and positive effects indicate the resistance allele is contributed by Mp705. ^‡^A negative dominance effect indicates that dominance is in the direction of the aflatoxin-reducing allele, regardless of the parental source, and a positive dominance effect indicates that dominance is in the direction of the aflatoxin-increasing allele. Epistatic interactions: A × A, additive × additive effect; D × D, dominance × dominance effect.*

Quantitative trait loci genetic effects were estimated by MIM that allowed for the identification of beneficial or aflatoxin-reducing alleles. In every environment, both parents contributed the beneficial allele, but the aflatoxin-reducing alleles have been passed down more stably from Mp719 in all environments (Table 5). When data was combined over all environments, Mp719 (resistant parent) contributed most beneficial alleles. The largest effects of each individual environment were found in bin 1.06 (additive, −0.294) in MS 2017, bin 3.07 (additive, −0.304) in TX 2017, bin 3.09 (additive, −0.304) in MS 2018, and bin 1.06 (additive, −0.274) combined across all environments and the source of the beneficial allele was Mp719 in every case.

## Discussion

Aflatoxin accumulation in maize is greatly influenced by the environment and the genetic background (Wang et al., 2019). The current study included the resistant parent, Mp719, the susceptible parent, Mp705, and their F1, planted as checks in each environment. The parents of the F2:3 population showed significant differences in aflatoxin cumulation when the data was transformed in the MS 2017 and MS 2018 environments. The F1 closely resembled Mp719, the resistant parent, but this is likely due to heterosis. For the F2:3 families, The MS 2017 environment had the lowest mean aflatoxin concentration compared to MS 2018 and TX 2017. Additionally, the coefficient of variability (standard deviation relative to the mean) was slightly higher for this environment. It is not clear if the difference in variance for MS 2017 compared to MS 2018 and TX 2017 was due to weather patterns. A mixed linear model was constructed and yielded the BLUPs for the F2:3 families. The mixed model also yielded estimates for the variance components and these were used to calculate the mean broad-sense heritability. The heritability of aflatoxin contamination in this study was low to moderate in agreement with those previously reported by Busboom and White (2004) and Brooks et al. (2005). Low heritability, as well as, high genotype-by-environment interaction, and the highly quantitative nature of this trait, has made the transfer of resistance to elite cultivars difficult to achieve.

Contamination of maize grain with aflatoxin has major economic implications and negative health consequences (Strosnider et al., 2006). The development of host-plant resistance as an approach to reduce aflatoxin contamination in maize has been met with challenges owing to the highly quantitative nature of this trait. Quantitative trait loci mapping used to identify closely-linked molecular markers, is proposed to aid in genetic improvement through marker-assisted breeding programs. Several bi-parental QTL mapping studies for resistance to aflatoxin accumulation have been conducted and QTL across all maize chromosomes have been detected. However, most of these mapping studies have a limited number of molecular markers covering the maize genome mainly because more highly dense maps were harder to come by in the past. In this study, a linkage map of Mp705 x Mp719 was mapped with a considerable amount of coverage at a relatively low resolution with average interval between markers of 1.3 cM. This genome coverage leads to precision QTL mapping needed for marker-assisted breeding.

Traditionally, plant breeders have utilized QTL-MAS for maize line improvement. This method has been used to introgress favorable alleles into an elite background. It was shown to speed up breeding efforts compared to conventional breeding methods. Marker-assisted selection based on genomic selection (GS) has been highlighted as a more novel approach in maize breeding in the last decade (Meuwissen et al., 2001). In GS, individuals with the most favorable estimated breeding values, which can lead to the identification of individuals harboring favorable alleles, are selected (Meuwissen et al., 2001). Both methods require high-density genetic markers; but, “GS-MAS requires a higher number of markers adequately covering the entire genome resulting in higher genotyping cost for GS-MAS” (Platten et al., 2019).

The current study identified two QTL in bins 1.06–1.07 (116.6–141.4 cM) and 3.09 (156.7–159.9 cM) (Supplementary Figure S1) that had the largest and most consistent genetic effects. The QTL on chromosome 3 was not in the region where makers were deviating from the Mendelian 1:2:1 ratio in bins 3.00–3.06. The aflatoxin reducing allele for these QTL came from Mp719, the resistant parent. When combined over all environments, these QTL, together, explained 24.6% of the phenotypic variance and 15.4% was contributed by the individual QTL found on 1.06. Because Mp719 was developed from the cross Mp715 × Va35, Mp719 likely acquired its aflatoxin-reducing alleles from its parents. In previous bi-parental QTL mapping studies involving Mp715, QTL were also found on chromosomes 1 and 3, and the aflatoxin reducing-alleles came mostly from Mp715 (Warburton et al., 2011; Dhakal et al., 2016; Smith et al., 2019). In a QTL mapping study of the bi-parental population Mp715 × Va35, Smith et al. (2019) reported a QTL on the short arm of chromosome 1 that was contributed by Va35, the susceptible parent of Mp719, and this QTL was consistently the source of the beneficial alleles in that region.

Breeding for a highly quantitative trait such as maize resistance to aflatoxin accumulation has been met with difficulties. Depending on the environment, multiple QTL in a common background must be used to reduce aflatoxin accumulation. Traditional breeding approaches complemented with QTL identification and associated molecular markers present an opportunity to increase understanding into the genetic basis of aflatoxin accumulation resistance in maize. Results in this study suggest that the aflatoxin-reducing QTL in the chromosomal regions bin 1.06 and 3.09 are critically important for developing aflatoxin-resistant maize lines and hybrids and should be the primary targets for transferring from Mp719 or Mp715 to elite lines with marker-assisted breeding.

## Data Availability Statement

All datasets generated for this study are included in the article/Supplementary Material.

## Author Contributions

EW and WW designed and planned the experiments. GW and WX performed the phenotyping. WW, GW, and WX contributed substantially to the writing and critical revision of the manuscript and approved its final version.

## Conflict of Interest

The authors declare that the research was conducted in the absence of any commercial or financial relationships that could be construed as a potential conflict of interest.
