# Supplementary material for: Mapping Quantitative Trait Loci Associated With Resistance to Aflatoxin Accumulation in Maize Inbred Mp719
Source: Front Microbiol. 2020 Feb 4;11:45. doi: 10.3389/fmicb.2020.00045 (PMC7010907; doi:10.3389/fmicb.2020.00045)
Supplement: Supplementary file 1 [file Image_1.pdf]

Supplemental Figure 1: LOD profiles for chromosomes 1 and 3 containing major QTL included in the final multiple interval mapping model of data combined over all three environments

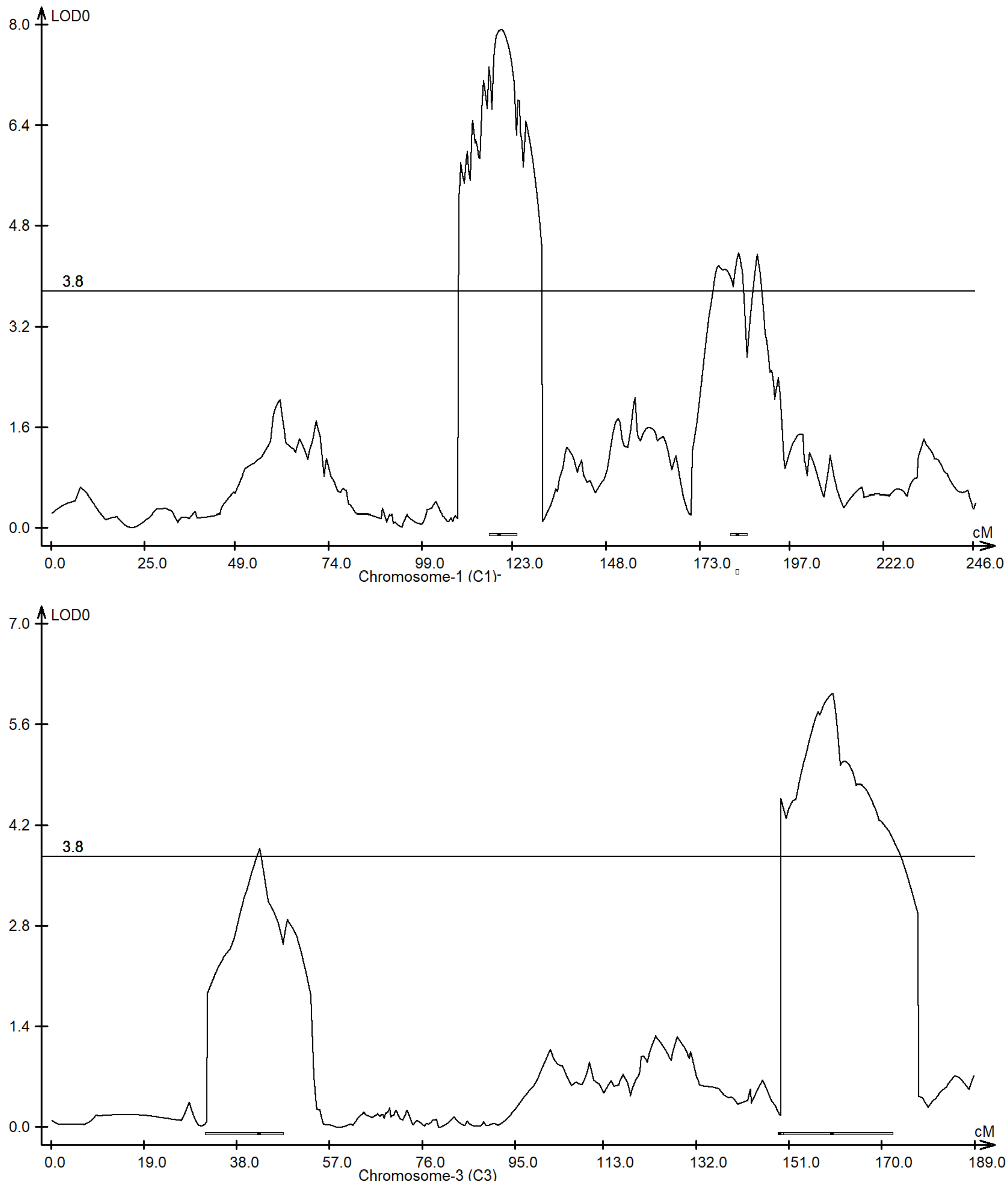

The LOD (y-axis) is a measure of the likelihood of a QTL being present at each test position along the chromosome (x-axis). The horizontal line across the figure is the LOD threshold. The x-axis has markers (not shown) mapped along its axis and is measured in centiMorgans (cM).
