# Supplementary material for: Mapping Quantitative Trait Loci Associated With Resistance to Aflatoxin Accumulation in Maize Inbred Mp719
Source: Front Microbiol. 2020 Feb 4;11:45. doi: 10.3389/fmicb.2020.00045 (PMC7010907; doi:10.3389/fmicb.2020.00045)
Supplement: Supplementary file 3 [file Table_2.docx]

Supplemental Table S2: Composite interval mapping results within and across environments

| Environment† | Chr Bin | QTL Peak Position | 2-LOD INTERVAL | LOD | LOD Threshold |
| --- | --- | --- | --- | --- | --- |
|  |  | **———————— cM ————————** | |  |  |
| MS 2017 | 1.06 | 124.4 | 124 - 125.8 | 5.9 | 3.8 |
|  | 1.10 | 183.2 | 175.7 - 185.4 | 4.1 |  |
|  | 8.01 | 17.4 | 14.5 - 18.2 | 3.8 |  |
|  |  |  |  |  |  |
| MS 2018 | 3.09 | 160.9 | 157.2 - 174.9 | 4.9 | 3.8 |
|  |  |  |  |  |  |
| TX 2017 | 1.07 | 141.8 | 140.1 - 145 | 4.0 | 3.8 |
|  |  |  |  |  |  |
| Combined | 1.06 | 119.7 | 117.4 - 124 | 7.9 | 3.8 |
|  | 1.10 | 183.2 | 181.8 - 185.4 | 4.4 |  |
|  | 2.06 | 85.7 | 85.3 - 88 | 4.0 |  |
|  | 3.03 | 42.6 | 31.9 - 47.3 | 3.9 |  |
|  | 3.09 | 149.2 | 149.1 - 150.3 | 4.6 |  |
|  | 3.09 | 159.9 | 150.3 - 172 | 6.0 |  |

†Environments: MS, Mississippi; TX, Texas
